# Circular RNAs as Therapeutic Agents and Targets

**DOI:** 10.3389/fphys.2018.01262

**Published:** 2018-10-09

**Authors:** Lesca M. Holdt, Alexander Kohlmaier, Daniel Teupser

**Affiliations:** Institute of Laboratory Medicine, University Hospital, Ludwig Maximilian University of Munich (LMU), Munich, Germany

**Keywords:** circRNA, transcription, splicing, microRNA sponges, aptamer, innate immnuity

## Abstract

It has recently been reported that thousands of covalently linked circular RNAs (circRNAs) are expressed from human genomes. circRNAs emerge during RNA splicing. circRNAs are circularized in a reaction termed “backsplicing,” whereby the spliceosome fuses a splice donor site in a downstream exon to a splice acceptor site in an upstream exon. Although a young field of research, first studies indicate that backsplicing is not an erroneous reaction of the spliceosome. Instead, circRNAs are produced in cells with high cell-type specificity and can exert biologically meaningful and specific functions. These observations and the finding that circRNAs are stable against exonucleolytic decay are raising the question whether circRNAs may be relevant as therapeutic agents and targets. In this review, we start out with a short introduction into classification, biogenesis and general molecular mechanisms of circRNAs. We then describe reports, where manipulating circRNA abundance has been shown to have therapeutic value in animal disease models *in vivo*, with a focus on cardiovascular disease (CVD). Starting from existing approaches, we outline particular challenges and opportunities for future circRNA-based therapeutic approaches that exploit stability and molecular effector functions of native circRNAs. We end with considerations which designer functions could be engineered into artificial therapeutic circular RNAs.

## Introduction

High-throughput RNA-sequencing has shown that at least 20% of presently active genes express circRNAs (Salzman et al., 2012; Jeck et al., 2013; Memczak et al., 2013; Guo et al., 2014; Jeck and Sharpless, 2014; Wang et al., 2014). CircRNAs are 3′-5′ covalently closed RNA rings, and circRNAs do not display 5′Cap or 3′poly(A) tails. 3–10 different circRNA isoforms are formed per host gene, resulting in tens of thousands of distinct circRNAs per cell type (Jeck et al., 2013; Memczak et al., 2013; Salzman et al., 2013; Guo et al., 2014; Westholm et al., 2014; Zhang et al., 2014; Ivanov et al., 2015; Rybak-Wolf et al., 2015). circRNAs are produced by the process of splicing, and circularization occurs using conventional splice sites mostly at annotated exon boundaries (Starke et al., 2015; Szabo et al., 2015). For circularization, splice sites are used in reverse: downstream splice donors are “backspliced” to upstream splice acceptors (see Jeck and Sharpless, 2014; Barrett and Salzman, 2016; Szabo and Salzman, 2016; Holdt et al., 2018 for review). The majority of circRNAs are 3′-5′-linked circles containing exons without intervening introns. On average, circRNAs contain 1–5 exons and are 500 ribonucleotides (nts) long (Jeck et al., 2013; Memczak et al., 2013; Salzman et al., 2013). Most circRNAs tend to be enriched in the cytoplasm, but notable exceptions exist (Salzman et al., 2012; Hansen et al., 2013; Jeck et al., 2013; Memczak et al., 2013; Liang and Wilusz, 2014; Zheng et al., 2016; Legnini et al., 2017). In 20% of all circularization events, introns do remain in the mature circRNA, constituting the class of Exon-Intron-containing-circular-RNAs (EIciRNAs) (Li et al., 2015). An even smaller fraction of circRNAs contains only introns and no exons, termed circular intronic RNAs (ciRNAs), which do not stem from backsplicing, but from conventionally spliced-out intronic lariats (Zhang et al., 2013). EIciRNAs and ciRNAs are defined by their presence and function in the nucleus (see chapter on molecular effector mechanisms below) (Zhang et al., 2013; Li et al., 2015). Given their circular nature, circRNAs of all classes are particularly stable compared to many linear RNAs because they are resistant to exonucleolytic decay by the cellular exosome ribonuclease complex (Szabo and Salzman, 2016; Holdt et al., 2018). The average half-life of endogenously produced 3′-5′-linked circRNA was found to amount to 19–24 h (Enuka et al., 2016) and can be up to 48 h (Jeck et al., 2013). In contrast, linear mRNAs show an average lifetime of only 4–9 h (Schwanhausser et al., 2011). High stability in biological systems is a major criterion for why circRNAs are becoming interesting for RNA-centered medical applications. Simultaneously, circularity is associated also with some other molecular properties that may be useful for therapeutic purposes, as will be discussed in the following.

## Biogenesis of Endogenous circRNAs

Details on circRNA biogenesis are described in recent reviews (Jeck and Sharpless, 2014; Barrett and Salzman, 2016; Szabo and Salzman, 2016; Holdt et al., 2018). In this chapter, we only highlight major points that are relevant for the subsequent considerations regarding therapeutic potential.

Splicing not only makes the linear transcriptome more diverse, but splicing also creates a very diverse set of circular RNAs. There is a tendency toward higher circRNA formation in more highly expressed genes, but overall, circRNAs are produced cell-type specifically (Salzman et al., 2013) and independent from changes in expression of their linear cognate transcripts (Salzman et al., 2013; Conn et al., 2015; Rybak-Wolf et al., 2015; Kristensen et al., 2018). To what extent circRNAs are regulated by global effects on backsplicing or circRNA stability during cellular transitions or due to gene-specific effects will have to be determined (Bachmayr-Heyda et al., 2015; Rybak-Wolf et al., 2015; Kristensen et al., 2018).

Analyses in different animal models have consistently shown that forward splicing is dominant, but backsplicing frequency is favored depending on context and availability of splice sites (Salzman et al., 2012; Ashwal-Fluss et al., 2014; Guo et al., 2014; Liang and Wilusz, 2014; Starke et al., 2015; Zhang et al., 2016; Liang et al., 2017): Two fundamentally different modes of circRNA biogenesis have been described: (1) Cotranscriptional backsplicing within the linear pre-mRNA and (2) Posttranscriptional backsplicing from within already excised exon(s)- and intron(s)-containing lariats (Barrett et al., 2015; Zhang et al., 2016). Intra-lariat backsplicing occurs physically separated from the maturing linear mRNA molecule. This distinction is of importance in functional terms, because only the execution of co-transcriptional backsplicing has been found to affect linear host mRNA from which the circRNA derives: After more than half of co-transcriptional backsplice events, the linear mRNA lacking the circularizing exon(s) cannot be detected anymore (Jeck et al., 2013). Also, there is a global anti-correlation of mRNA levels and circRNA formation frequency, supporting the view of mutual competition between linear splicing and backsplicing (Ashwal-Fluss et al., 2014; Zhang et al., 2014; Koh et al., 2016).

The following parameters determine circRNA biogenesis (relating to both co- and post-transcriptional modes):

(A) Reverse complementary repeats in the flanking introns, for example of the family of Alu repeats in the human genome, favor the backsplice reaction by hybridizing with each other and bringing splice sites in close proximity (Dubin et al., 1995; Liang and Wilusz, 2014; Zhang et al., 2014; Ivanov et al., 2015).
(B) A rather specialized compendium of splicing factor and RNA-binding proteins partakes in backsplicing through positioning splice sites in 3-dimensional space in the nucleus (Fujita et al., 1988; Ho et al., 2004; Ashwal-Fluss et al., 2014; Conn et al., 2015; Kramer et al., 2015; Errichelli et al., 2017; Li X. et al., 2017). This includes SR-family and hnRNP-family splicing regulators (Kramer et al., 2015; Li X. et al., 2017), and factors like Quaking (Conn et al., 2015), Muscleblind (Ashwal-Fluss et al., 2014) and FUS (Errichelli et al., 2017).
(C) The efficiency of backsplicing increases when forward splicing was kinetically slowed-down, such as after linear mRNA polyadenylation was impaired and read-through into adjacent genes occurred, or after core-spliceosomal components, such as the SF3a/b complexes, were limited in promoting the forward splicing reaction (Liang et al., 2017).

**FIGURE 1:**
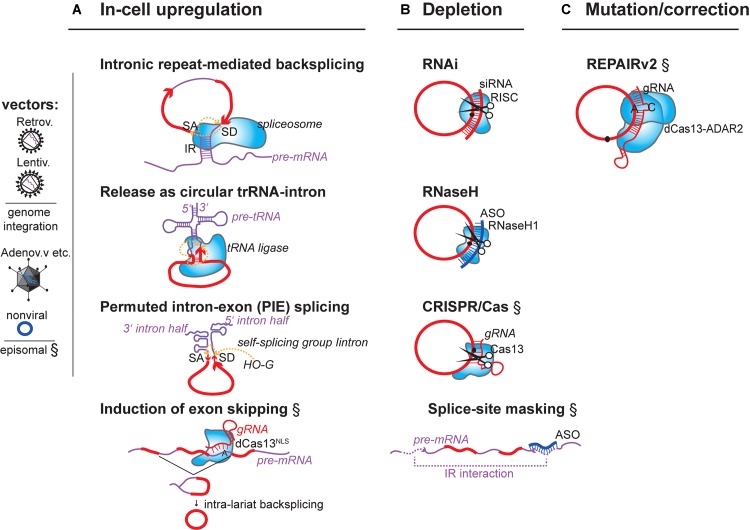
Summary of concepts for modulation of endogenous circRNAs in mammalian cells. Strategies that have experimentally not yet been achieved are marked (§). **(A)** circRNA-overexpression using mini-gene constructs on RNA-/DNA vectors or affecting the endogenous circRNA-generating locus. (i) DNA mini-gene constructs contain RNA polymerase II (RNAP II) transcription start and terminator sites, splice acceptor (SA) and splice donor sites (SD). Exon(s) in the mini-gene constructs are >300 nucleotides (nts) long (Barrett et al., 2015; Kramer et al., 2015) and are flanked by intronic inverted repeats (IR) (Zhang et al., 2014) that are >30–40nts (Liang and Wilusz, 2014) and up to 300–500 nts long (Hansen et al., 2013; Kramer et al., 2015). (ii) RNA circularization through tRNA intron splicing. Expression by U6 promoter and capping signal (Lu et al., 2015). (iii) RNA circularization through autocatalytic intron-group I self-splicing. Catalysis still occurs when the catalytic intron is split into two halves, and when the order of introns and exons is inverted (3′ intron and SA upstream of 5′ intron and SD), but in such (permutated) cases, different from normal group I splicing, the intervening exonic sequence (arrow) in the PIE construct becomes circularized (Puttaraju and Been, 1992). G-OH (free guanosine) serving as hydrophile that initiates transesterifications. (iv) Enhancing circRNA levels by increasing the frequency of circRNA biogenesis. When exon-skipping is increased, more circRNAs can be produced that derive from skipped exons through post-transcriptional intra-lariat splicing. Targeting the catalytically inactive nuclear dCas13 variant via guide RNAs to intronic branchpoint adenosines and/or splice donor sites is known to increase skipping (Konermann et al., 2018). **(B)** circRNA depletion strategies: Depletion of mature circRNAs by (i) the shRNA-/siRNA loaded RISC complex (top), (ii) by RNase H endonuclease-dependent degradation. RNase H can be recruitment to circRNAs via transfected DNA-like antisense oligonucleotides (ASO, middle), or (iii) by single-stranded-RNA-targeting CRISPR/Cas nuclease versions (Class 2 CRISPR/Cas13a, b, d). An alternative is to (iv) block circRNA biogenesis through masking intronic inverted repeats (IRs) via complementary ASOs (bottom). **(C)** Mutating/Correcting circRNA sequence: Expressing a fusion protein consisting of the ADAR2 adenosine deaminase linked to the catalytically inactive dCas13 nuclease allows deamination of A > I (inosine) at sites specified by a mismatch (C)-containing guide RNA that locates dCas13 to a specific circRNA. Inosine will base-pair with cytidine, potentially changing the properties of the circRNA at this site (changing binding affinities to other RNAs, DNAs or protein). Fusion of dCAs13 to different RNA-editing enzymes will expand the range of possible ribonucleotide conversions. RNA (red), introns (purple), antisense oligos (dark blue), RNA-binding proteins (light blue shapes).

How circRNA biogenesis occurs in detail is a matter of ongoing research, and also bioinformatic circRNA-mapping algorithms are still improving (Szabo and Salzman, 2016; Hansen, 2018). Most DNA constructs that have been successfully employed for circRNA overexpression in cells used parts of native introns with inverted repeats (IRs) therein, or employ sequences artificially cloned in reverse complementary orientation adjacent to circularizing exons (**Figure 1A**) (Zhang et al., 2014; Kramer et al., 2015; Starke et al., 2015).

## Molecular Effector Mechanisms of circRNAs

Details of known circRNA-effector mechanisms and functions in physiology and disease are described in recent reviews (Jeck and Sharpless, 2014; Barrett and Salzman, 2016; Szabo and Salzman, 2016; Holdt et al., 2018; Patop and Kadener, 2018). Briefly, circular RNAs have been found to be biologically active agents in two conceptually distinct ways: either as freely diffusible molecules in nucleus and cytoplasm or because the circRNA biogenesis process itself has functional implications. Five major functions are known altogether:

(1) Circular RNAs may influence RNA polymerase II (RNAP II) initiation and elongation of their host gene. As best understood cases, subgroups of EIciRNAs (Li et al., 2015) and ciRNAs (Zhang et al., 2013), amounting to approximately 100 circular RNAs in each class, have been found to bind to RNAP II at promoters and to stimulate transcription by molecularly mostly unclear effector pathways. For example, EIciRNAs *circEIF3J* and *circPAIP2* associated with promoters depending on the U1 non-coding RNA (Li et al., 2015), which is known from separate work to instruct and activate general transcription initiation and elongation factors TFIIH and P-TEFb in the context of RNAP II transcription initiation and elongation.
(2) The co-transcriptional biogenesis of 3′-5′-linked circRNAs can reduce linear host mRNA levels and change downstream splice-site choice in mRNAs by still unknown mechanisms (Jeck et al., 2013; Ashwal-Fluss et al., 2014; Zhang et al., 2014; Koh et al., 2016): This has been described for endogenous candidate circRNAs, such as *circMbl* (Ashwal-Fluss et al., 2014) or *ASXL1* (Koh et al., 2016), but evidence suggests this mechanism is of genome-wide significance. One possible determinant for competition with linear splicing is that backsplicing inflicts a 2′-5′ knot in the linear mRNA (Jeck and Sharpless, 2014; Barrett et al., 2015), possibly leading to termination of downstream transcription or serving as an entry point for exonuclease-mediated mRNA degradation (Liang et al., 2017). A second point is that backsplicing correlates positively with faster RNAP II transcription speed on gene bodies (Zhang et al., 2016), which *per se* affects splice site choice, an effect known as kinetic coupling between transcription and splicing apparatus (Caceres and Kornblihtt, 2002; de la Mata et al., 2003; Ip et al., 2011; Dujardin et al., 2014; Fong et al., 2014). Overall, backsplicing is thought to impair linear mRNAs not so much when circRNAs are posttranscriptionally produced, that is when they arise by backsplicing from within already excised exon-containing lariats (Barrett et al., 2015). Yet, also circRNAs are known that can affect linear mRNAs splicing choice as *trans*-acting factors, as shown for *circSEP3* in the plant *Arabidopsis thaliana* (Conn et al., 2017a).
(3) Few circRNAs, including *CDR1as* and Sry, carry a higher density of microRNA binding sites. These sequester and functionally inactivate microRNAs, an effect termed “sponging” (Hansen et al., 2013; Memczak et al., 2013). While *CDR1as* and *Sry* encode 74 and 16 of microRNA seeds, and are sufficiently highly expressed compared to the number of microRNA targets in a cell, as well as compared to the absolute number of microRNA copies per cell, few other endogenous circRNAs are expected to fulfill the stoichiometric requirements to be functional circRNAs at endogenous level (Mullokandov et al., 2012; Bosson et al., 2014; Denzler et al., 2014, 2016; Karreth et al., 2015; Thomson and Dinger, 2016). Also, compared to exons in mRNAs that do not circularize, circRNA-included exons are not overrepresented in Argonaute 2 (AGO2) pull-downs, nor are microRNA seed-matched sites overrepresented near backsplice junctions (Guo et al., 2014; You et al., 2015). Thus, the classical sponging concept seems inflated in currently published studies of endogenous circRNAs. The situation may be different for circRNAs whose therapeutic usefulness resides in their overexpression, as discussed below.
(4) Some mature circRNAs can bind to proteins to sequester them or affect their activity, like *circMBL* binding to MBL protein, *circPOLR2A, circDHX34* binding to NF90 and NF110, *circANRIL* binding to PES1 and *circPAPBN1* binding to HuR (Ashwal-Fluss et al., 2014; Holdt et al., 2016; Abdelmohsen et al., 2017; Li X. et al., 2017). CircRNAs can also serve as a scaffold for protein complexes, such as *circFOXO3* binding to CDK2/p21 (Du et al., 2016).
(5) At the moment, the view prevails that the vast majority of the thousands of circRNAs is not found in an active translation state (Jeck et al., 2013; Bazzini et al., 2014; You et al., 2015). Most circRNAs do also not stem from the very 5′ regions of genes (Guo et al., 2014), and, therefore, besides the fact that they do not carry a linear 5′Cap structure, do not encode features that enable protein translation from an endogenous start codon. A tiny fraction (<1%) of circRNAs does happen to contain the start AUG codon and to be associated with active ribosomes. In these cases circularization had led to the inclusion of 5′ untranslated region (5′UTR) sequences. For a handful of tested circRNAs, like *Drosophila circMbl, circPde8*, *circTai, circCdi* and mouse *circZNF609*, these 5′UTRs structurally folded into internal ribosome entry sites (IRES). This allowed the circRNAs to associate with the small subunit of ribosomes and become templates for protein translation despite being circular (Legnini et al., 2017; Pamudurti et al., 2017). Besides IRES-mediated translation, N(6)-adenosine methylation (m6A) has recently been shown to promote 5′Cap-independent translation in linear mRNAs (Zhou et al., 2015). This modification is also observed on circRNAs (Legnini et al., 2017; Zhou et al., 2017). But any specific role of m6A for circRNA translation, among many other possible influences of this modification on RNA half-life, sorting, and structure, remains to be decisively determined.

## circRNA Manipulation in CVD Disease Models *in Vivo*

The molecular analysis of circRNAs is still in its infancy, but it is becoming clear that circRNAs can be functionally relevant, and that functions can be quite diverse. In the following chapter, we will focus on those circRNAs whose therapeutic potential can be assessed from knockdown or circRNA overexpression in mouse disease models of CVD *in vivo* (**Table 1**).

**Table 1:** Therapeutic potential of circRNAs in CVD.

Name (Species)	Investigated disease	Expression in disease	Endogenous function	Target cells (indirect evidence)	Protection from disease through	Mouse models *in vivo*	Dose; route	Reference
*MFACR*	Myocardial infarction	Up	Deleterious	Cardiomyocytes?	circRNA KD	Injection of adenovirus expressing shRNAs against *MFACR* into the aortic root rescues from heart dysfunction (I/R-induced heart injury mouse model).	2 × 10^11^ Pfu; once per 14 days	Wang et al., 2016, 2017
*HRCR*	Heart failure (cardiac hypertrophy)	Down	Protective	Cardiomyocytes?	circRNA OE	Injection of *HRCR*-generating adenovirus into jugular vein rescues from cardiac hypertrophy (ISO-induced heart injury mouse model).	2 × 10^11^ Pfu; once per 14 days	Wang et al., 2016
*circAmotl1*	Heart failure (Cardiomyopathy)	n.d.	Protective	Cardiomyocytes?	circRNA OE	Intraperitoneally injection of nanoparticles with mPEG-SH-formulated DNA plasmid (pBS) expressing human *circAmotl1* ameliorates ventricle dilation and stress-induced cell death (dox-induced mouse cardiomyopathy model).	100 mg plasmid; 2-3x per week	Zeng et al., 2017
*circFoxo3*	Heart failure (senescence, cardiomyopathy)	Up	Deleterious	Cardiomyocytes, fibroblasts?	circRNA KD	Injection of PEG-(10-nm) gold nanoparticle with siRNAs against *circFoxo3* ameliorates doxorubicin-induced cardiomyopathy.	6 μg, 3 times/week	Du et al., 2017b
*circHipk3*	Diabetic retinopathy	Up	Deleterious	Retinal vascular endothelial cells?	circRNA KD	Intravitreal injection of adenovirus-encoded shRNAs targeting *circHIPK3* in a mouse model for diabetic retinopathy is protective.	1.5 × 10^9^ Pfu; once per month	Shan et al., 2017
*cZnf609*	Ischemic retinopathy	Up	Deleterious	Retinal vascular endothelial cells?	circRNA KD	Intravitreal injection of adenovirus-encoded shRNAs against *cZNF609* ameliorates retinopathy in diabetic and hyperoxia-induced mouse retinopathy models.	1 × 10^7^ Pfu; once-twice per week	Liu et al., 2017
*circDlgap4*	Stroke	Down	Protective	Brain endothelial cells?	circRNA OE	Injection of *circDLGAP4*-generating lentivirus ameliorates brain damage in a transient middle cerebral artery occlusion mouse model.	5 × 10^8^ Pfu; once per 2 weeks	Bai et al., 2018


*List of studies where levels of circRNAs have been manipulated with a therapeutic success in animal CVD models in vivo. CVD, cardiovascular disease; I/R, ischemia/reperfusion; ISO, isoproterenol; KD, knockdown; mPEG-SH, thiol-functionalized methoxyl polyethylene glycol; OE, overexpression; Pfu, plaque forming unit; shRNA, short hairpin interfering RNA.*

### Myocardial Infarction

The mouse circRNA *MFACR* (acronym for *mitochondrial fission and apoptosis-related circRNA*) was identified to be upregulated in a mouse model of heart ischemia/reperfusion (Wang et al., 2017). *MFACR* was implicated as a sponge for *miR-652-3p*, a microRNA that targets MTP18, a nuclear-encoded mitochondrial protein important for balanced mitochondrial fission and cell viability. *MFACR* has 15 seed-matched sites for *miR-652-3p*. In a heart injury model *in vivo*, systemic inhibition of *MFACR* reduced apoptosis and heart dysfunction (Wang et al., 2017). Thus, a future therapeutic intervention would be to deplete *MFACR* after injury or to protect *miR-652-3p* from being sequestered by using conventional linear DNA/RNA oligonucleotides. Metabolically active cardiomyocytes may be particularly susceptible to this mechanism (**Table 1**).

### Heart Failure

The mouse circRNA *HRCR* (acronym for *heart-related circRNA*) was found to be downregulated during experimentally-induced cardiac hypertrophy (Wang et al., 2016). Having six seed-matched binding sites for *miR-223*, *HRCR* was implicated as *miR-223* sponge. This microRNA targets the mRNA transcribed from the *ARC* gene (*Apoptosis repressor with CARD domain*) and protects from hypertrophy in injured hearts. Systemic *HRCR* overexpression in a mouse heart failure model inactivated *miR-223*, hence, activating its target ARC. This also curbed cardiac hypertrophy. Since human *miR-223* levels were increased and ARC levels decreased in failing human hearts, a therapeutic option would be to deliver *HRCR* or to inhibit its downregulation (**Table 1**).

*circ-Amotl1* was enriched in the neonatal (regenerative) heart compared with mature hearts in humans (Zeng et al., 2017). Systemic overexpression of human *circ-Amotl1* ameliorated left ventricle dilation and reduced apoptosis in a mouse cardiomyopathy model (Zeng et al., 2017). As an effector mechanism, the authors suggested that *circ-Amotl1* physically bound to well-known pro-growth and pro-survival AKT-PDK1 protein complexes in the phosphoinositide-3-kinase–protein kinase signaling pathway and activated them. *circ-Amotl1* would have to be therapeutically delivered with caution, however, because it has been independently described to be tumorigenic (Zeng et al., 2017) (**Table 1**). Whether the therapeutic effect in the rodent disease model is due to the physical interaction of human *circ-Amotl1* with the mouse AKT-PDK1 complex is still not fully clear. It should also be noted in this context, that human *circ-Amotl1* is not conserved in mouse or rat, although triggering consistent cellular effects. At least theoretically one can expect that the structural conservation of the AKT and PDK1 proteins are sufficiently high in mammals to allow *trans*-species interaction of circRNA with these proteins, but this remains to be shown.

Chemotherapeutic drugs and other stressors trigger senescence-associated pathological changes in hearts, and *circFoxo3* was studied as aging-induced circRNA (Du et al., 2017b). When siRNAs against *circFoxo3* were administered, the pathological left ventricular dilation in a doxorubicin-induced cardiomyopathy mouse model decreased, cardiac fibrosis was ameliorated and apoptosis and senescence-associated β-Galactosidase accumulation decreased (Du et al., 2017b). Conversely, overexpression of *circFOXO3* from mini-gene constructs on injected plasmids worsened heart function parameters. Determining the underlying molecular pathway will be complex, not last, because the described function cannot easily be tied to the known role of *circFoxo3* as more general cell cycle inhibitor (Du et al., 2016). Also, Foxo3 protein produced from linear *Foxo3* mRNA is implicated in cell growth suppression in the heart and other organs, and in senescence onset and longevity control (**Table 1**).

### Neoangiogenesis

*circHIPK3* is induced in diabetic human retinas (Shan et al., 2017). When systemically depleting *circHIPK3* with shRNAs targeting the backsplice junction in a mouse model for diabetic retinopathy, the disease phenotype was ameliorated. Complicating a direct therapeutic use, *circHIPK3* activity confers a general pro-proliferative function in a number of different cell types (Zheng et al., 2016). Additionally, *circHIPK3* downregulation increases migration, invasion, and angiogenesis of cancer cells (Li Y. et al., 2017), and impairs β-cell function (Stoll et al., 2018). Therefore, a system-wide and long-term downregulation of *circHIPK3* may carry an increased risk for cancer development and metabolic syndrome (**Table 1**).

*circZNF609*, a proposed *miR-615-5p* sponge, was shown to be induced in diabetic human retinas. *circZNF609* contains one seed-matched binding site. The mouse orthologous circRNA exerted deleterious cell overproliferation and a pro-angiogenic role while expressing shRNAs against *cZNF609* reduced capillary degeneration and pathological angiogenesis in a mouse oxygen-induced retinopathy model (Liu et al., 2017). The accessibility of the eye for topically administering interfering oligonucleotides might make *cZNF609* an accessible candidate for therapy. At the same time, *cZNF609* carries separate functions in myoblast differentiation (Legnini et al., 2017), raising issues on the specificity of effect (**Table 1**).

### Stroke

*circDLGAP4*, a proposed *miR-143* sponge, was less abundant in brain tissue in a rodent cerebral stroke model, and in blood plasma of human acute ischemic stroke patients (Bai et al., 2018). The circRNAs has one seed-matched binding site for *miR-143*. Injecting *circDLGAP4-*generating lentivirus into the lateral brain ventricle reduced the ischemic infarct volume and improved neurological function in the mouse stroke model (**Table 1**).

The studies described here are representative of how circRNAs are currently used as therapeutic agents and targets in different pathologies. It should be understood that the presented cases are published studies related to CVD, and not necessarily those with the highest expectable therapeutic potential. In all cases, circRNAs and circRNA-knockdown construct are delivered systemically, although lesions are local, and although the candidate circRNAs are known to be endogenously expressed in more than one organ. It is, therefore, rather astonishing, but also promising, that such interventions were able to ameliorate specific CVD endpoints at all. It must be stated that before clinical trials could be thought of, the expected negative effects on proliferation and differentiation of non-target cells and organs have to be addressed.

## Stability of Circular RNAs in Biological Systems

Stability against exonucleases is an overarching feature of circRNAs. Highly stable therapeutic RNAs could be valuable in cases where the therapeutic agent must be administered less frequently, or in smaller doses, which would also minimize non-specific side effects. Endogenously produced circRNAs are 2-5 times more stable than linear RNAs (Schwanhausser et al., 2011; Jeck et al., 2013; Enuka et al., 2016). What these numbers mean for ectopically delivered therapeutic ribonucleic acids is not so clear, yet. No data exist that report on the stability of circRNAs *in vivo*. A single study, so far, reported on the stability of synthetic circRNAs in cultured cells *in vitro* (Jost et al., 2018): Their half-life was 8–20 h, depending on the delivery regime, which can be considered short compared to endogenous circRNAs and to the times of effectiveness of modern linear RNA therapeutic agents. For example, antagomiRs, modified antisense DNA- or RNA-like oligonucleotides (ASOs) and double-stranded siRNAs all show *in vivo* effects for 10–15 days, and up to several weeks, after a single injection into the body (Bennett and Swayze, 2010; Crooke et al., 2018; Jost et al., 2018). In analogy to ASOs, and with the aim to improve stability especially in the extracellular space, circRNAs could in the future be engineered to carry, for example, 2′-*O*-methyl-, -fluoro- or –*O*-methoxyethyl conjugates, phosphorothioate backbones, or 2′,4′-cyclic 2′-*O*-ethyl modifications (Krutzfeldt et al., 2005; Crooke et al., 2018). However, some types of modifications are thought to trigger liver and renal toxicity or local cutaneous reactions (Swayze et al., 2007; Burdick et al., 2014; Burel et al., 2016). On the other hand, chemical modification of circRNAs may theoretically optimize also other pharmacokinetic properties, binding to targets and escape from immune sensors.

## Future Therapeutic Avenues Using circRNAs

A future therapeutic use of circRNAs could be envisioned in two ways. One is the modulation of native disease-linked circRNAs by therapeutic knockdown or by ectopic expression. A second is the engineering of non-native (artificial) circRNAs with designer molecular effects (**Figure 1A**).

### Modulation of Native Disease-Linked circRNAs

**FIGURE 2:**
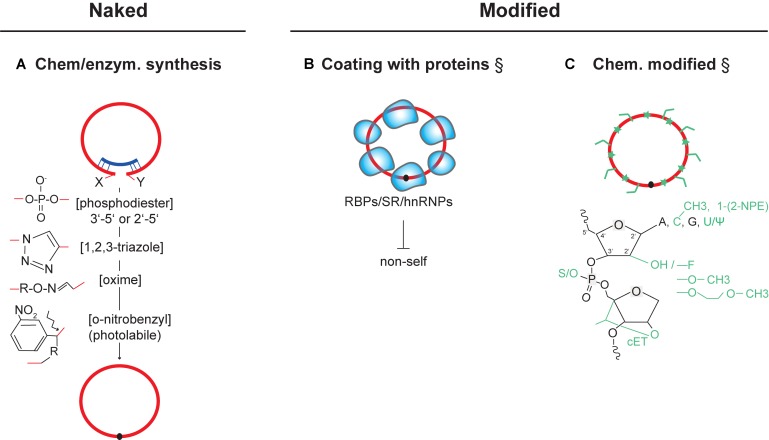
Delivery of *in vitro* circularized circRNAs for therapeutic use. Strategies that have experimentally not yet been achieved are marked (§). **(A)** Chemical structures of key *cis*-reactive chemical moieties mediating chemical or enzymatic circularization (by RNA ligases or ribozymes). As one example, introducing photolabile linkers into the backbone of circular RNAs can be employed to acutely inactivate circRNAs through linearization (Richards et al., 2010; Griepenburg et al., 2013; Wu et al., 2013) (bottom). **(B)** Strategy for coating synthetically produced circRNAs with RNA-binding proteins and splicing factors. Coating could be used to avoid recognition as non-self and inhibit innate-immune activation after transfection. **(C)** Possible chemical modification of synthetic circRNAs at RNA bases, 2′ position in the ribose, and in the phosphodiester backbone. Modifications can have effects on improving stability, binding affinity, and pharmacokinetics (see text). ψ (Pseudouridine), cET (2′,4′-constrained ethyl), 1-[2-NPE] (1-[2-nitrophenyl]ethyl). NPE-like modifications of the RNA nucleobases, or other photolabile cages in RNA, can be conditionally cleaved-off by light, allowing acute conversion to an active state (Schafer et al., 2013). RNA (red), antisense oligos (dark blue), RNA-binding proteins (light blue shapes).

At least two concepts for therapeutic modulation of endogenous disease-linked circRNAs are conceivable: (1) Modulation of native circRNAs (**Figure 1**) and (2) Engineering of *in vitro*-produced circRNAs (**Figure 2**). To modulate native circRNAs, one can overexpress native protective circRNAs and reconstitute missing circRNAs from genetic vectors (**Figure 1A**), deplete endogenous disease-promoting circRNAs (**Figure 1B**), or correct aberrantly produced circRNA-isoforms (**Figure 1C**). On the other hand, *in vitro*-produced RNA circles can be transferred into cells as unmodified RNA (**Figure 2A**) or as modified RNA (**Figure 2B**).

Overexpression of native protective circRNAs has been achieved from RNAP II-driven constructs on standard DNA expression vectors in cell culture (Hansen et al., 2013; Memczak et al., 2013; Ashwal-Fluss et al., 2014; Li et al., 2015; Du et al., 2016; Holdt et al., 2016; Legnini et al., 2017), and from lentiviral or adenoviral vectors *in vivo* (Wang et al., 2017; Bai et al., 2018; Xia et al., 2018) (**Figure 1A**, see also **Table 1**). These encode a mini-gene cassette with exon(s), endogenous splice donor and acceptor sites, and flanking intronic inverted repeats that support RNA backfolding (**Figure 1A**). Alternatively, and independently of the cellular spliceosome, circRNAs are successfully expressed also from within intron boundaries encoded from the anticodon stem-loop on an engineered tRNA construct (Salgia et al., 2003; Lu et al., 2015; Noto et al., 2017). Processing, in this case, occurs by the evolutionarily conserved tRNA ligase complex (RTCB/HSPC117 ortholog in humans (Popow et al., 2011)) (**Figure 1A**). In-cell production of human circRNAs can be engineered to involve RNA ribozymes that mediate circularization by RNA self-processing (Zaug et al., 1983; Sullivan and Cech, 1985; Puttaraju and Been, 1992; Wesselhoeft et al., 2018) (**Figure 1A**). Another tangible approach for post-transcriptional production of circRNAs would be to use ASOs that bind to splice sites or splice enhancers and increase the frequency of co-transcriptional backsplicing. This would enhance the primary alternative splicing event so that more circularization can occur when more exon-containing lariats are present (**Figure 1A**). Given the competition with linear splicing (Jeck et al., 2013; Ashwal-Fluss et al., 2014; Jeck and Sharpless, 2014; Zhang et al., 2014, 2016; Barrett et al., 2015; Koh et al., 2016; Liang et al., 2017), the same strategy would indirectly also decrease the levels of cognate linear host mRNAs. Since the underlying competition is thought to occur *in cis*, at the level of pre-mRNA splicing, and not via *trans*-acting circRNAs, such a re-programming of mRNA levels would not be possible by delivering circRNAs via plasmids or transfecting synthetic circRNAs. Since backsplicing amounts, however, to only a low percentage (>10%) of the total mRNA output of any protein-coding gene (Chen et al., 2017), the potency of such trans-interference will be accordingly low. This can, however, also be an advantage and allow fine-tuning of the expression landscape.

Depletion of endogenous disease-promoting circRNAs has already been achieved using standard genetic tools, RNA interference (RNAi) or ASO-mediated RNase H-dependent degradation (see Swarts et al., 2014; Khvorova and Watts, 2017 for recent reviews) (**Figure 1B**). In the future, modern ssRNA-targeting by CRISPR-Cas9/13 variants (O’Connell et al., 2014; Abudayyeh et al., 2016, 2017; Konermann et al., 2018) will likely be applied (**Figure 1B**). To achieve specificity in circRNA-knockdown, all these nucleases must be guided selectively to the circRNA-specific backsplice-junction (Barrett et al., 2017; Piwecka et al., 2017). In the literature, *MFACR*, *circHIPK3*, *cZNF609*, and *circFoxo3* (**Table 1**) have been depleted *in vivo* using short hairping (shRNAs) and small interfering siRNAs to trigger RNAi. An already implemented and very specific therapeutic approach for circRNA knockdown relates to depletion of fusion-circRNAs (f-circRNAs): These arise when chromosomal translocations bring introns from two unrelated genes in close genomic vicinity, which undergo backsplicing (Guarnerio et al., 2016). It was recently shown that knockdown of these f-circRNAs was sufficient to trigger apoptosis in leukemic cells while no toxic effects on normal cells could be measured (Guarnerio et al., 2016). Thus, f-circRNAs are interesting and potentially rather selective targets in treating certain cancers and, maybe, also other translocation-triggered pathologies. A parallel option to decrease circRNA levels would be to administer ASOs that bind and mask regulatory splice enhancers or silencers, or inverted intronic repeats in a specific pre-mRNA before circRNA biogenesis can take place (**Figure 1B**). This has not yet been experimentally tested. As mentioned before, inhibition of backfolding should in theory block circularization, and in some instances even favor linear mRNA splicing, given the competition between co-transcriptional backsplicing and mRNA maturation.

Not last, being able to sequence-specifically correct ribonucleic acid sequence inside living cells will, likely, become another interesting option also for circRNA-based therapeutics (**Figure 1C**): Fusion of catalytically-inactive Cas13 enzyme with an ADAR deaminase has already been proven successful for manipulating linear RNA sequence at the level of the transcript (Cox et al., 2017).

Delivering synthetic RNA circles into cells is another therapeutic approach. Such synthetic circRNAs are produced *in vitro* and must then be transfected into target cells (**Figure 2**). Different methods exist to achieve RNA circularization (see Muller and Appel, 2017 for review) (**Figure 2A**) and to formulate such circRNAs, such as by coating them with native RNA-binding proteins (**Figure 2B**), or by adding chemical moieties that change the physicochemical properties of the RNA (**Figure 2C**). While the consequences of artificial circularizing linkers have to be addressed functionally in each case, first estimation allow to predict that their structural and chemical alterations tend to be minor (e.g., regarding circularization by 2′-5′ bonds (Sheng et al., 2014) or by triazole linkers (El-Sagheer and Brown, 2012)] (**Figure 2A**). Among different circularization strategies, methods using ribozymes and click-chemistry-based linkage seem to be particularly efficient and cost-effective alternatives to commercial recombinant RNA ligases.

### Engineered Non-native (Artificial) Circular RNAs with Designer Molecular Effects

**FIGURE 3:**
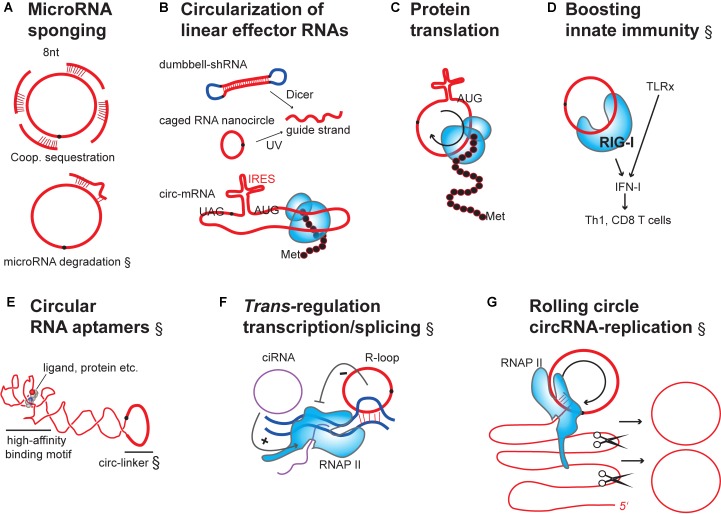
Engineering designer effector functions in overexpressed or transfected circRNAs. **(A)** circRNAs as microRNA sponges: Binding of microRNAs to circRNAs containing multiple, cooperatively-acting seed-matched sites leads to inactivation by sequestration (top). Additional bonding via the 3′end of microRNAs can lead to target-induced decay of the microRNA (bottom). **(B)** Artificial circularization of linear effector RNAs: linear effector RNAs can be circularized to increase the potency of effect (e.g., shRNAs, siRNAs or mRNA). For siRNAs to become active, their precursors need to be processed (top). mRNAs must be engineered to contain an internal ribosome entry site (IRES) for protein translation (bottom). **(C)** Rolling-circle translation from circRNAs: In the absence of stop codons continuous translation can occur, leading to repetitive polypeptides. These can have potential structural and functional use, or be toxic. **(D)** Naked circRNAs can be delivered to purposefully boost the innate immune system and, indirectly, influence quality and quantity of effector and memory lymphocyte adaptive immunity. TLRx, Toll-like receptors; IFN, interferon; Th1, T helper cells; CD8 T cells, cytotoxic T cells. **(E)** Use of circular RNAs as stable aptamers: schematic of circular RNA aptamer binding to a ligand/protein by adopting a high-affinity structure (G-quadruplex motif) (Huang et al., 2014). Redrawn from the crystal structure of the *Spinach* RNA aptamer bound to its ligand (PDB (Berman et al., 2002): 4Q9Q). Note that the circularizing linker was not in the original structure. **(F)** Stimulation of transcription initiation and elongation by different circular RNAs species binding to RNAP II: Intron-only circular RNAs (ciRNAs) bind to promoter regions: exon-only 3′-5′ circRNAs can bind to gene loci through triple-helix formation (R-loop). circRNAs of this class would hypothetically be useful for fine-tuning of transcription, epigenetic engineering, or encoding synthetic transcriptional circuits. **(G)** Hypothetical model for self-replication of synthetically produced and transfected circRNA: Permanent circRNA maintenance in dividing cells involves host cell RNAP II-dependent rolling-circle RNA transcription, self-splicing of the long linear multimeric RNA precursor into single linear RNA units and circularization by host cell RNA ligases. RNA (red), introns (purple), DNA or DNA linkers (dark blue), RNA-binding proteins (light blue shapes).

Aside from modulating the expression of native circRNAs, circRNAs can also be engineered to carry specific properties (**Figure 3**). Here, we will discuss several major concepts for engineering designer circRNAs, with the most feasible ones ranked first: (A) MicroRNA-sponging by circRNAs; (B) Circularization of known linear therapeutic RNAs; (C) Protein translation from circRNAs; (D) Modulation of the immune system by circRNAs; (E) Protein activity control by circular RNA aptamers; (F) Transcription/splicing control by circRNAs; and (G) Engineering autonomous RNA replication of therapeutically delivered circRNAs *in vivo*.

(A) MicroRNA sponging by hybridization to 8 nts-long seed motifs in competing-endogenous RNAs, including circRNAs, prevents homologous target mRNAs from degradation or allows their translation (Franco-Zorrilla et al., 2007; Poliseno et al., 2010; Cesana et al., 2011; Salmena et al., 2011; Tay et al., 2011) (**Figure 3A**). Considering recently published experiments, only a few endogenous circRNAs like *circCDR1as* or *circSry* (Hansen et al., 2013; Memczak et al., 2013) would meet the very specific conditions and stoichiometric requirements to be endogenous microRNA sponges (Mullokandov et al., 2012; Bosson et al., 2014; Denzler et al., 2014, 2016; Karreth et al., 2015; Thomson and Dinger, 2016). Yet, it can be expected that the act of overexpressing a therapeutic circRNAs changes stoichiometry and allows these circRNAs to function as microRNA sponges. Such overexpressed circRNAs could even be designed to be better sponges by closely spacing microRNA seeds, which is expected to trigger cooperative microRNA-inhibition (Grimson et al., 2007; Saetrom et al., 2007; Schirle and MacRae, 2012; Pfaff et al., 2013). However, to be efficient, it has been calculated that circRNA abundance would have to be sufficiently high relative to the number of microRNA seed-targets in mRNAs (Mullokandov et al., 2012; Bosson et al., 2014; Denzler et al., 2014, 2016; Karreth et al., 2015; Thomson and Dinger, 2016) and close to the target mRNA abundance (Yuan et al., 2015). Another consideration has to be taken into account: Recently, 6 nts-long seeds have been found to trigger microRNA-degradation instead of mere sequestration (Denzler et al., 2016). This opens another therapeutic avenue for circRNAs. Already, one study has reported that an artificial circRNA had been successfully overexpressed that was able to sponge the liver-specific *microRNA-122* (Jost et al., 2018). The engineered circRNA sponge contained 8 seed-matched sites for *miR-122*, and these were separated by 4 nts spacers (Jost et al., 2018). This circular RNA construct inhibited *miR-122* in its known role as stimulator of translation of the human *Hepatitis C RNA-Virus* in a cell culture model (Jost et al., 2018). The inhibitory efficiency *in vitro* was significant and comparable to the effect of the drug Miravirsen, a short linear locked-nucleic-acid (LNA) that targets the virus by complementary binding to *miR-122* (Jost et al., 2018).
(B) Given their stability against exonucleases, one tempting option would be to circularize classically employed linear RNAs, such as the small siRNA precursors or the sense strand of siRNAs (**Figure 3B**). This is possible, because compared to the rigid dsDNA helix (Egli et al., 1990), the flexibility of RNA molecules allows circularization of even very small RNAs. But also longer linear mRNAs and long non-coding RNAs (lncRNAs) could hypothetically be circularized (Kool, 1998; Abe et al., 2007, 2008, 2011). Still, one would have to test whether basic interaction parameters, such as binding to other nucleic acids and proteins, would still be intact in such articificial circles.
  A promising case is the circularization of mRNA and their use for stable protein translation in target cells (**Figure 3B**, bottom). As described above, more than 99% of endogenous circRNAs are never productively associating with ribosomes, and in no case, so far, has the function of a circRNA-templated polypeptide been documented. Therapeutically it is, however conceivable, that exons encoded in engineered circRNAs could be translated to proteins when the ribosome is capture via an IRES, and when a complete open reading frame with stop and start codon were included through engineering (Chen and Sarnow, 1995). Therefore, mRNA circularization could be useful for therapeutic consideration since it has been shown that circularity is no principle obstacle for translation through the ribosome, once the ribosome has been recruited through an engineered IRES (Chen and Sarnow, 1995; Wang and Wang, 2015) (**Figures 3B,C**). Such a strategy could be interesting for many linear RNAs/mRNAs that are already therapeutically used, for gene expression control, vaccination, or as aptamers (see existing reviews for example of linear therapeutic RNAs (Sullenger and Nair, 2016; Matsui and Corey, 2017; Crooke et al., 2018; Lieberman, 2018). A recent proof-of-concept study has shown that translation from circularized mRNA sequences is a viable option for therapeutic protein expression and is even superior to translation from linear transcripts (Wesselhoeft et al., 2018): mRNAs of up to 5 kilobases were shown to be efficiently circularized when transcribed in mammalian cells from rationally-designed DNA vectors encoding a self-splicing group I intron from a cyanobacterial (*Anabaena*) pre-tRNA as ribo-enzymatic factor. Translation was particularly strong when using unconventional IRES elements, such as from the Coxsackievirus B3, and when adding structural spacing elements between IRES and splice sites. Comparing to state-of-the-art translation from transfected linear mRNAs with 5′Cap, 3′polyA tail and internal 5′methoxyuridine modifications for stabilized expressed, translation from unmodified circRNAs drove 50% more protein and provided protein over a two-times longer time period (80–120 h after a single RNA transfection) (Wesselhoeft et al., 2018).
(C) Care has to be taken about the specificity of translation from circRNAs. In the absence of in-frame stop codons and termination signals, or after read-through, rolling-circle-translation can occur on circular RNAs. Rolling-circle translation can lead to translation of multimeric repetitive protein motifs, which could be useful for cell engineering, but which can become toxic for cells if happening in an uncontrolled fashion (Jeck and Sharpless, 2014; Abe et al., 2015) (**Figure 3C**).
(D) In another consideration, linear RNAs have been shown to be therapeutically useful as adjuvants for boosting immune therapy (Le Bon et al., 2003; Hochheiser et al., 2016) (**Figure 3D**). In this pathway, RNA-sensing in the cytoplasm triggers innate immunity, and innate immune signaling is known to contribute to adaptive immunity by diverse routes. One option would, thus, be to use circRNAs as tools to purposefully boost innate immune signaling to counteract disease-induced immunosuppression, or as adjuvants for boosting adaptive immunity during vaccination. This is not far-fetched as circularity of RNA has recently been shown to be sensed by the RNA-binding RIG-I molecules, which trigger downstream innate immune signaling (Chen et al., 2017). One way ahead would be to introduce generic circRNAs (not targeting any host mRNA) which carry RNA-sequence or dsRNA-forming motifs known from independent work to activate the RIG-I (Hwang et al., 2012; Schnell et al., 2012) and RIG-I-like receptors (Pippig et al., 2009), which might increase circRNA-dependent immunity even further.
(E) Another therapeutic option would be to employ circRNA-based RNA aptamers that bind with high specificity, for example, to proteins (even those without RNA-binding domains) (**Figure 3E**). Among many other options, aptamers are useful for selective drug delivery or protein activity control. Circularizing them might make them more potent. circRNAs have already before been suggested to be potentially useful to affect the availability and subcellular sorting of RNA-binding proteins RBPs (Hentze and Preiss, 2013), many of which are already known to function in physiology and disease, not last through impacting linear mRNA splicing (see Holdt et al., 2018 for review). Technically, SELEX (“*Systematic Evolution of Ligands by EXponential Enrichment”*) has been devised as a powerful unbiased PCR-based method for molecularly improving binding or effector mechanism *in vitro* for linear aptamer nucleic acids (Ellington and Szostak, 1990; Tuerk and Gold, 1990). Many modern variations of this technique exist (see Darmostuk et al., 2015 for review), and SELEX would be adaptable, in principle, for circular RNa aptamer evolution *in vitro*. In fact, a recent attempt has succeeded to produce circular RNA aptamers (Umekage and Kikuchi, 2009). Circular RNA aptamers can be screened totally *de novo*, but in might also base on native sequences known to confer binding to a range of proteins (Ashwal-Fluss et al., 2014; Kramer et al., 2015; Li et al., 2015, Du et al., 2016, 2017a,b; Holdt et al., 2016; Schneider et al., 2016; Abdelmohsen et al., 2017; Chen et al., 2017; Li X. et al., 2017). To date, no specific protein-interaction motif in a circRNA has been analyzed in any detail. But the fact that circular RNAs are constraint in secondary structure compared to linear RNAs, has let to speculations that circular aptamers may perform even better in terms of specificity and binding affinity (Lasda and Parker, 2014).
(F) Many endogenously expressed 3′-5′-linked circRNAs are thought to be modulators of transcription and splicing of linear mRNA, mostly of cognate linear transcripts produced from the circRNA host gene. The modulation happens when backsplicing occurs cotranscriptionally within pre-mRNA (Zaphiropoulos, 1996; Lewis et al., 2003; Suzuki et al., 2006; Jeck et al., 2013). Specific cases of *trans*-regulatory circular RNAs exist, and these spur the hope to be able to exploit this mechanism for therapeutic purposes (**Figure 3F**): For example, the class of intron-only ciRNA is thought to influence genomic loci in *trans*, whereby ciRNAs bind (RNAP II) and boost transcription (Zhang et al., 2013). Independently, at least one classical 3′-5′-linked exonic circRNA has been shown to affect linear splicing when provided posttranscriptionally *in trans* (Conn et al., 2017b). It was found to hybridize to complementary sequences in the host DNA locus, forming a triple helix (termed R-loop), which might be causal for impairing RNAP II elongation and RNAP II-coupled splicing. It has so far not been shown whether either of these *trans*-regulatory effects on transcription can be engineered into artificial circRNAs in therapeutic applications (**Figure 3F**).
(G) To date, no tools exist that would allow circRNA replication after transfection of synthetic RNA into mammalian cells. RNA replication mechanisms that do not rely on the integration of DNA intermediates into the genome exist in nature (**Figure 3G**). For example circular RNA viroids in plants are replicated as RNAs *in vivo*. This occurs by an RNA-rolling-circle mechanism, whereby host DNA-dependent RNA-polymerase is repurposed for transcription of the RNA circle (Muhlbach and Sanger, 1979; Navarro et al., 2000). After transcription, the linear transcripts are cleaved into monomers by host RNase II-type enzymes (Gas et al., 2008) or self-encoded hammerhead-ribozymes (Hutchins et al., 1986). Finally, unit-length RNAs are circularized by host RNA-/DNA-/tRNA ligases (Gas et al., 2008; Nohales et al., 2012). Importantly, also the single-stranded RNA genome of the human hepatitis-delta-virus replicates as RNA *in vivo*, speculatively by an RNA-rolling-circle-mechanism (Taylor, 2015). For RNA-therapeutics, it will be paramount to explore whether RNA-replication could be artificially transferred from any of the known physiological systems onto an engineered synthetic circRNA (of minus-polarity) and exploited for long-term maintenance of effector RNA circles (plus-polarity). This would be particularly relevant for maintaining circRNA that are to be transfected into dividing cell types, where circRNA levels quickly drop by continued dilution.

## Therapeutic Delivery of circRNAs

A longstanding problem inherent to the therapeutic use of nucleic acids has been its route of application. The delivery of circRNAs follows, in principle, existing methods for delivering therapeutic RNAs, as no specialized possibilities or physicochemical obstructions have yet been found associated with circularity *per se*. Delivery strategies involve systemic injection into the vasculature, subcutaneous injection or depots, or local application. Obviously, strategies to achieve delivery to receptors on specific target organs through chemically functionalizing therapeutic RNAs (e.g., with *N*-acetylgalactosamine), cannot use end-modification in a circular RNA. Instead such modifications would have to be introduced, for example, by functionalizing the ribose 2′ hydroxyl group in the circRNA backbone. A lot of work has been dedicated to achieve tropism in atherosclerosis therapy by using homing peptides or fatty acids, or functionalized RNAs and lipid carriers that target only atherosclerotic plaques (Hamzah et al., 2011; Hofmeister et al., 2015; Deshpande et al., 2016; Huang et al., 2016; Zhang J. et al., 2017). For CVD therapy, local delivery at sites of atherosclerotic lesions includes stent-coating with polymers and hydrogels containing and releasing therapeutic RNAs (Koenig et al., 2017). Also, modern transfection methods could be adaptable to deliver circRNAs during cardiovascular operations, including photo-/optoacoustic approaches, which allow defining target cells and delivering cargo via nanostructures (Zhang Y. et al., 2017).

Typically, individual nucleotides can freely enter cells, while circRNAs and circRNA-generating vectors are insufficiently hydrophobic and too large (>1000 Daltons) to passively pass the cell membrane’s phospholipid bilayer (Lipinski et al., 2001; Mansy et al., 2008). Instead endogenously expressed circRNAs, as other linear RNAs, are thought to exit the circulation and enter target cells via endocytosis (Juliano et al., 2014). Developing strategies for therapeutic circRNA delivery to cells via lipid carriers and for exiting from the endosomal membrane after cellular uptake will, therefore, directly benefit from the existing experience with transfecting nucleic/ribonucleic acids, naked or complexed with proteins, via traditional cationic and lipidic transfection routes, ionizable lipids and modern lipid, synthetic and functionalized nanoparticles (see Crooke et al., 2017, 2018; Dowdy, 2017 for review). In the end, despite emerging insight into the nuclear export mechanism for circRNAs (Huang et al., 2018), is not yet known how circular RNAs could be delivered to the nucleus, or how the nuclear exit to the cytoplasm could be controllable, which restrains some of the therapeutic considerations.

## Potential Side Effects of circRNAs Modulation

As with any therapy, it is anticipated that modulation of circRNAs might also have side effects. The knockdown of circRNAs suffers from the same potential off-targeting effects as for linear mRNA interference, aggravated by the fact, that knockdown must specifically target the unique circRNA back-splice junction and is, thus, even more, constrained (Barrett et al., 2017; Piwecka et al., 2017). Also, the ectopic expression of circRNAs from DNA vectors is potentially problematic: circRNAs biogenesis has been found not to be uniform in all cell types, and, besides, running-circle transcription from the DNA and production of linear RNA concatemers can occur. Concatemers can be toxic, and be substrates for linear splicing, which can lead to uncontrollable biological effects (Barrett and Salzman, 2016). While synthetic circRNAs might be preferred, for this reason, their mass synthesis and delivery are more problematic. Concerning delivery, synthetic circRNAs, unless coated through RBPs, are sensed as “non-self” and trigger RIG-I-dependent innate immune signaling upon cell entry (Umekage and Kikuchi, 2009). This can confound analysis and therapy. Immunodetection might, in the future, be circumvented by masking circRNAs with recombinant proteins that would usually be loaded on circRNAs by endogenous backsplicing (Umekage and Kikuchi, 2009), or by chemical RNA pseudo-uridination, as has already described for linear ribonucleotides (Kariko et al., 2005; Durbin et al., 2016).

It should also be noted that for all cases of therapeutic circRNAs described in **Table 1**, it has not yet been explored, whether the therapeutic effect was due to the change in circRNA level in the diseased target tissue. Especially for CVD, disease parameters might change as a consequence of the systemic effects of circRNAs on liver function, metabolism or the immune system. In this case, superior methods may deliver the therapeutic circRNAs more directly to those primary effector organs and increase the therapeutic potency.

## Outlook

Form today’s perspective, it is well conceivable that circRNAs may serve as therapeutic agents and targets in the near future. CircRNAs might either be modulated intracellularly or administered as *in vitro* synthesized formulations. As of now, it is still difficult to synthesize circRNAs at a sufficient scale in a cost-effective manner. *In vitro* synthesis of circRNAs is currently still performed with recombinant enzymes, which is costly when substantial amounts are required for therapy. Thus, alternatives for routine high-scale synthesis are being developed, such as allosterically regulated ribozymes, which perform circularization but are unable to re-cleave the circular RNA end-product and, thus, are envisioned to increase circRNA yield (Muller and Appel, 2017). Different hybrid approaches are conceivable, where large amounts of mammalian circRNA could be produced in yeast, which is cultivatable in industrial scale and genetically easy to program (Hossain and Johnson, 2014; Wang et al., 2014). This is an issue, as genetically impairing the RNA-processing machinery has recently been shown to be a valid option to increase backsplicing (Liang et al., 2017). If and how this can be achieved without compromising cell growth is still unknown.

Overall, it has taken 4 decades to overcome some of the major hurdles for using linear RNA as therapeutics. Since technological insight is in many cases directly transferrable from linear therapeutic RNAs to circRNAs, the use of circRNAs as therapeutic agents and targets in human diseases is only a question of time and might occur earlier than expected.

## Author Contributions

All authors listed have made a substantial, direct and intellectual contribution to the work, and approved it for publication.

## Conflict of Interest Statement

The authors declare that the research was conducted in the absence of any commercial or financial relationships that could be construed as a potential conflict of interest.
